# Look at the lung: can chest ultrasonography be useful in pregnancy?

**DOI:** 10.1186/2049-6958-9-32

**Published:** 2014-06-06

**Authors:** Riccardo Inchingolo, Andrea Smargiassi, Flaminio Mormile, Roberta Marra, Sara De Carolis, Antonio Lanzone, Salvatore Valente, Giuseppe M Corbo

**Affiliations:** 1 Pulmonary Medicine Department, University Hospital “A. Gemelli”, Rome, Italy; 2 Obstetric Pathology Department, University Hospital “A. Gemelli”, Rome, Italy; 3 Pulmonary Medicine Department, Università Cattolica del Sacro Cuore, Rome 00168, Italy

**Keywords:** Chest ultrasound, Pregnancy, Thoracic imaging, Pneumonia, Pleural disease, Pregnancy, Thoracic imaging

## Abstract

**Background:**

This study aimed to evaluate the clinical value of chest ultrasound (US) in the detection, diagnosis and follow-up of pathologic processes of both peripheral lung parenchyma and pleural space in pregnant women.

**Findings:**

Pregnant women admitted to Obstetric Pathology Hospital Department for respiratory diseases were enrolled. Chest US examination was performed when there was a respiratory disease highly suggestive of pneumonia and/or pleural effusion and chest X-ray (CXR) should have been obtained. Three chest US patterns were identified: lung consolidation (LC), pleural effusion (PE) and focal sonographic interstitial syndromes (SIS). When chest US pathologic signs were reported, one or more subsequent chest US examinations were performed to follow-up the patient until their complete resolution.

Sixteen inpatients underwent 54 chest US evaluations. We identified: 9 LCs, 6 PEs and 11 SISs. Total number of CXRs was 7 (10 females avoided X-rays exposure and one underwent 2 CXR evaluations on the advice of Gynecologist). Chest US follow-up, during and after therapy, showed complete resolution of echographic patterns previously described.

**Conclusions:**

Chest US evaluation during pregnancy is a useful diagnostic tool to detect and monitor respiratory diseases, avoiding excessive X-rays exposure.

## Findings

### Introduction

The usefulness of ultrasound in pregnancy is approved as a low-risk diagnostic imaging technique if compared to ionizing radiations. The use of chest ultrasound (US) is gaining ever growing acceptance in order to detect pathological conditions of the respiratory tract [1-3]. As far as pleural effusions are concerned the use of chest ultrasounds has been reported in literature for many years [4] and as regards to pulmonary consolidations its usefulness is easily understandable [5-7].

Also the study of the horizontal and vertical artifacts allows an ever growing comprehension of these phenomena and their applications to clinical practice [3,8].

Chest US evaluation can be performed at patient bed-side and, added to clinical and anamnestic examination, can help physicians to get the correct diagnoses and guide eventual invasive procedures. Moreover, it can be also used to monitor pneumonia and pleural effusions during therapy until complete resolution [6,7]. Recently published meta-analysis supported chest US as an established diagnostic tool for the diagnosis of pneumonia, when performed by experienced physicians [8].

For these reasons, we tried to apply chest US evaluation in order to detect, diagnose and monitor pathologic processes of peripheral lung parenchyma and of pleural space in pregnant women.

In these cases ultrasound evaluation can be extremely appropriate especially if ionizing radiations can be avoided.

### Case series

We presented a series of cases of pregnant women affected by respiratory pathologies, detected, managed and followed up during therapies with the aid of chest ultrasonography.

Sixteen pregnant women, admitted for respiratory diseases to the Obstetric Pathology Department of University Hospital “A. Gemelli” in Rome, were enrolled. The study received approval by the Institutional Review Board. Patients were enrolled in a period of 24 months from January 2012. Each patient gave written informed consent. Mean pregnancy time was 27 weeks (±10), ranging from 11^th^ to 38^th^ week.

Patients underwent chest US examination when there was a respiratory disease highly suggestive of pneumonia or/and pleural effusion (i.e. fever, dyspnea, leukocytosis, cough, chest pain) [5].

Chest ultrasonography was performed by two pulmonologists (RI and AS), blinded to CXR findings. Chest US was performed after CXR. Moreover, an independent radiologist blinded to other findings interpreted all CXR. The interobserver agreement was evaluated.

For all procedures, MyLab™ 50 Gold Cardiovascular (Esaote S.p.a., Rome, Italy) machine with 3.5-5 and 7.5-10 MHz probes was used.

A 3.5-5 MHz convex probe was used to localize lung consolidation and pleural effusion collection with the patient sitting. A 7,5-10 MHz linear probe was used to detect detailed features of pleural line and to study eventual sonographic interstitial syndrome. Multiple scans were obtained in both sagittal and intercostal planes.

### Echographic patterns

**Figure 1:**
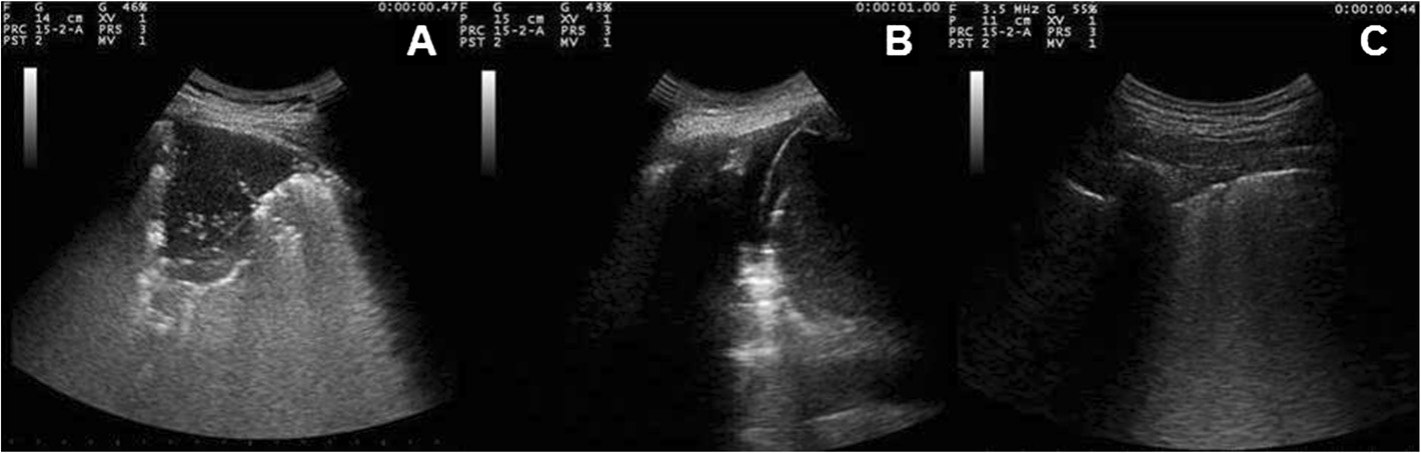
Chest ultrasonographic patterns: lung consolidation (A), pleural effusion (B) and sonographic interstitial syndrome (C).

We identified 3 kinds of pathologic chest US patterns: lung consolidation, pleural effusion and sonographic interstitial syndrome, as described in literature [1-3,9] (Figure 1 and Table 1). When chest US pathologic signs were reported, one or more subsequent chest US examinations were performed in order to follow up the patient until their complete resolution.

**Table 1:** Description of echographic patterns in chest ultrasonography

**Echographic patterns**	**Description**
Normal	Immediately below the chest wall planes, the presence of a regular and continuous hyperechoic line (pleural line) without any evidence of artifactual vertical images and with motionless and regularly spaced horizontal artefacts of reverberation
Lung consolidation	The evidence of well-delimitated subpleural (but surfacing in pleura) hypoechoic sonographic solid structures that are multiform in shape and at times involving whole lobes of the lung
Pleural effusion	Fluid of whatever nature (inflammatory, transudative, hematic, etc.) that accumulates in the pleural space causing a separation of the parietal and visceral layers of the pleura, appearing as a prevalently non-echogenic area that collects between the lung parenchyma and the chest wall
Focal sonographic interstitial syndrome	The presence of rare, dense, or confluent B-lines (hyperechoic narrowbased artifacts spreading like laser rays from the pleural line to the edge of the screen) or of white lung (completely white echographic lung field with or without merged B-lines and with no horizontal reverberation). Focal sonographic interstitial syndrome is topographically detectable only in relation to limited zones of pleuralparenchymal pathological alterations

**Figure 2:**
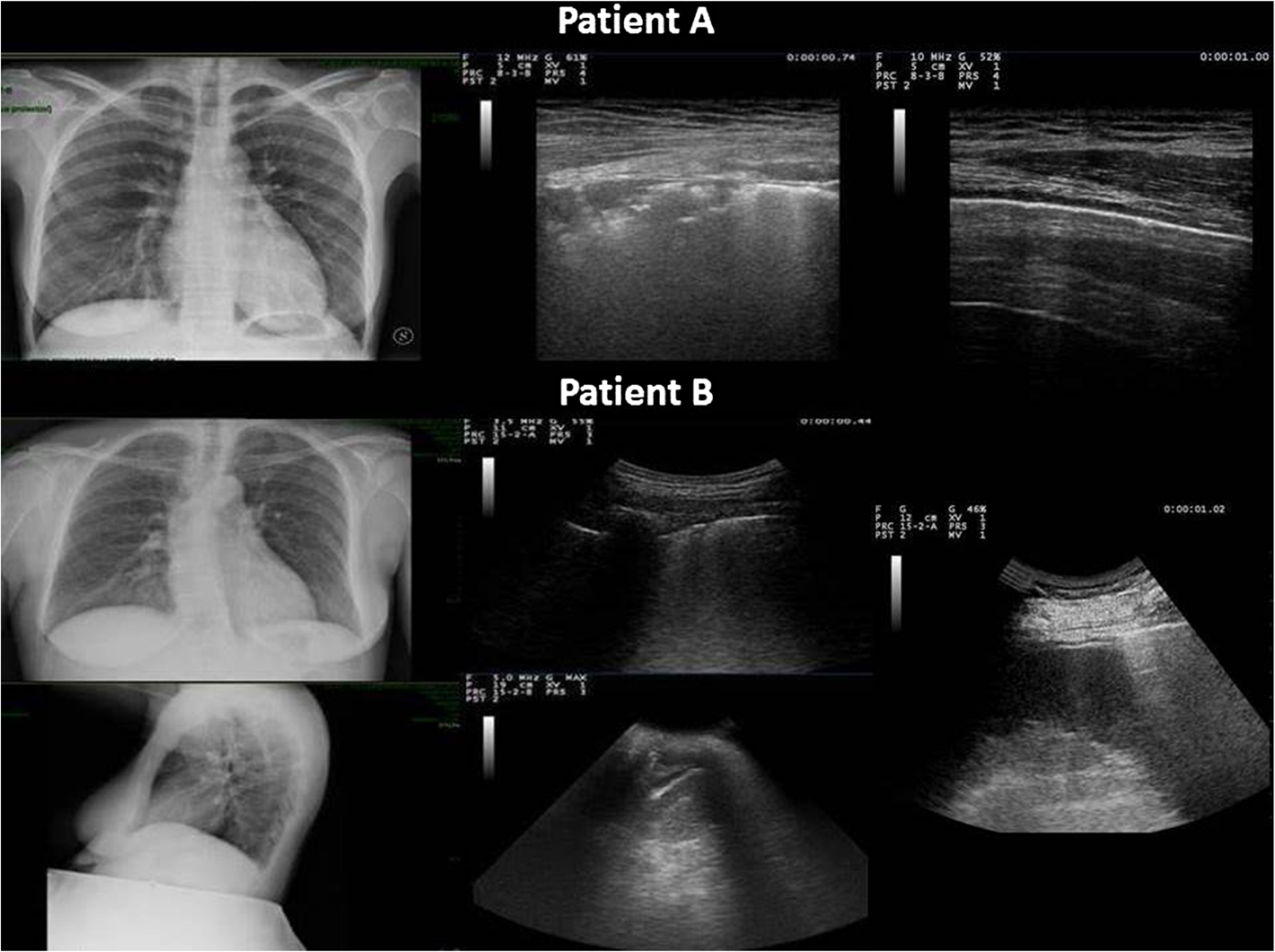
**Two cases of difficult to detect CXR pathologic signs managed by chest US.** Patient **A**: *On the left*: CXR reported difficult-to-detect pulmonary consolidation of the left lower lobe consistent with retrocardiac pneumoniax. *In the middle*: The ultrasound assessment with linear array probe shows delimitated subpleural hypoechoic solid structures surfacing in pleura. Focal pulmonary edema related to inflammatory effects is found near lung consolidations. These findings are associated with small lung consolidations consistent with pneumonia. *On the right*: Normalized Chest US pattern after therapy. Patient **B**: CXR performed in anterior-posterior (*at top left*) and lateral scans (*at bottom left*) did not show easily detectable pathologic findings. *In the middle*: The ultrasound assessment with convex probe showed focal echographic interstitial syndrome and focal alterations of the pleural line of a limited dorsal region of the left lower lobe (*at top*). A minimal free flowing left pleural effusion, limited to costo-phrenic sinus same-sided with the focal echographic interstitial syndrome (*at bottom*). *On the right*: Visualization of curtain sign on spleen without evidence of pleural effusion after therapy.

Echographic patterns suggestive of pulmonary and/or pleural pathologic involvement were reported in 12 patients. In particular, we identified 9 lung consolidations, 6 pleural effusions and 11 focal sonographic interstitial syndromes. Nine focal interstitial syndromes were associated to lung consolidation as peri-lesional pattern. Four pleural effusions were reported in association with lung consolidation and 1 in association with focal sonographic interstitial syndrome. Four patients did not show any pathologic chest US pattern. In these women pneumonia and pleural effusion were excluded and a final diagnosis of lower airway infection was made. Ten patients showed leukocytosis and 1 patient showed leukopenia. The overall number of chest US performed was 54. Six patients underwent chest X-ray (CXR) after clinical evaluation and on medical decision at admission in Emergency Department. CXR, when performed, showed pathological signs suggestive of pneumonia/pleural effusion in four patients; while, in two cases, the pleural/parenchymal involvement was difficult to detect as instead assessed by chest US (Figure 2 and Table 2).

**Table 2:** Characteristics of patients

**Patient**	**Focal sonographic interstitial syndrome**	**Lung consolidation**	**Pleural effusion**	**WBC**	* Chest X Ray	**N° of chest US**	**Gestation time**
1	+	+		16,71	+	3	37
2			+	10,24		3	16
3				16,43	+	3	36
4	+		+	15,47	+	3	34
5	+	+		13,22	+	3	24
6	+			3,41		3	28
7	+	+	+	18,54		3	30
8	+	+		9,73		3	38
9				7,54		3	38
10				9,68		3	32
11	+	+	+	8,36	+	5	11
12				23,96		3	38
13	+	+	+	24,81		3	11
14	+	+		6,75		3	25
15	+	+	+	24,49	+	4	18
16	+	+		15,43		6	15

+: if present.

*: white blood cells [*109/L].

All the pathologic echographic signs described and reported in this study changed towards a complete resolution in the last chest US evaluation, with all patients gradually getting better.

### Discussion

Diagnosing pneumonia in pregnancy has a strong clinical relevance. In fact, pneumonia can affect pregnant women determining health risks for mother and fetus. Community acquired pneumonia is the most common fatal non-obstetric infectious complication and a common cause of hospital admission [10]. It has been demonstrated that women with pneumonia during pregnancy had significantly higher risk of low birthweight, preterm birth, small-for-gestational age, cesarian section, lower Apgar score and pre-eclampsia/eclampsia compared to unaffected women [11].

As already reported in literature, the use of radiation for diagnostic imaging in pregnant women is associated with a high level of anxiety for the patient, her family and her doctors [12].

The effects of radiation exposure in pregnancy depend on the exposure time as well as fetal absorbed dose, and in very early gestational time is more likely dangerous for the conceptus in terms of failure to implant and undetectable death. So, non-urgent x-rays should be avoided in weeks 10–17 [13].

Pregnant women and fetus are considered to be particularly vulnerable to the adverse effects of radiation exposure and, when diagnostic radiography becomes necessary and unavoidable, the woman should be counseled regarding the benefits and the risks of the procedure, even if exposure radiations, not involving direct abdominal–pelvic high dosage, are associated with no significant adverse events [12].

Although there are no strong contraindications to perform diagnostic radiography, especially if not abdominal–pelvic anatomic districts are involved, it is beyond any doubt that this situation can cause stress in pregnant women and in doctors even if a counseling is performed.

Ultrasound imaging is widely used during pregnancy without any documented fetal or maternal adverse effects and this modality has largely replaced x-ray as the primary diagnostic tool for abdomen and pelvic districts.

Obviously inflated lungs are considered undetectable by ultrasound imaging because of their high acoustic impedance: lung ultrasonography has not been considered feasible for many years.

In case of inflated lungs, the ultrasound beam is reflected by the tissue/air interface (“mirror” phenomenon) [2].

However several conditions are able to alter the mirror phenomenon with the appearance of vertical artifacts [3]. They would be produced by complex and poorly understood acoustic interactions of the ultrasound beam with peripheral lung parenchyma when its airspace geometry and its porosity are altered as in pathologic conditions [9,14].

Focal sonographic interstitial syndrome may be observed as consequence of inflammatory effects near lung consolidations. Thus, when consolidation is not at direct contact with visceral pleural surface, some indirect signs may be recognized [3,5,6].

Chest ultrasound can be performed bed-side whenever required being well accepted by pregnant women. Moreover, its feasibility allows doctor to monitor and follow up pathological findings until complete resolution. All the ultrasound abnormalities were no longer detected at the final examination.

## Conclusion

This study clearly confirms the usefulness and the feasibility of chest ultrasonography in pregnancy. We do not propone at the current time the substitution of chest-x-ray with chest ultrasonography and this study does not want to compare the two imaging techniques. Further studies need to be made in order to completely validate this technique in the diagnosis of pregnancy-related pneumonia. However, it looks promising as a diagnostic and follow-up imaging technique especially because radiations might be avoided in several circumstances.

## Competing interests

The authors declare that they have no competing interests.

## Authors’ contributions

All Authors have equally 1) made substantial contributions to conception and design, or acquisition of data, or analysis and interpretation of data, 2) been involved in drafting the manuscript or revising it critically for important intellectual content, 3) given final approval of the version to be published. Finally, all Authors agree to be accountable for all aspects of the work in ensuring that questions related to the accuracy or integrity of any part of the work have been appropriately investigated and resolved.
